# Management of miscarriage: a matter of choice? A mixed- methods research study in Northern Ireland

**DOI:** 10.1186/s12978-026-02316-x

**Published:** 2026-03-13

**Authors:** M Galeotti, G Mitchell, M Tomlinson, Á Aventin

**Affiliations:** 1 School of Nursing & Midwifery, Queen’s University Belfast, 97 Lisburn Rd, Belfast, Northern Ireland BT9 7BL UK; 2 Department of Global Health, Institute for Life Course Health Research, Stellenbosch University, Cape Town, South Africa

**Keywords:** Management of miscarriage, Miscarriage treatment, Miscarriage, Pregnancy loss, Maternal mental health, Women’s mental health, Health and wellbeing

## Abstract

**Background:**

Miscarriage, defined in the United Kingdom as the loss of a pregnancy before 24 weeks’ gestation, remains the most common gestational complication, affecting 13–15% of all recognised pregnancies. Despite established NICE guidelines outlining three management options—expectant, medical, and surgical—little is known about how these approaches influence women’s emotional wellbeing or sense of autonomy. This mixed-methods study explored women’s experiences of miscarriage management and their emotional needs when attending hospital facilities in Northern Ireland (NI).

**Methods:**

A cross-sectional online survey and semi-structured interviews were conducted between January and April 2021. Participants (*N* = 723) were women aged over 16 years who had experienced a miscarriage in NI within the preceding five years. Quantitative data were analysed descriptively, while qualitative data were thematically analysed.

**Findings:**

Findings revealed that 42% of participants experienced expectant management, 14% medical, 28% surgical, and 16% a combination of treatments. Notably, 52% of respondents reported that they were not given a choice of management, with 36% stating that healthcare professionals made decisions without clear explanation. Among those who were not offered a choice, 40% were dissatisfied with the information received, and 44% felt insufficient time was allowed to discuss options. Conversely, women who were offered choice reported higher satisfaction, clearer communication, and greater perceived emotional support. Thematic analysis identified three central themes from the qualitative data: choice and autonomy, importance of being informed, and time for decision-making.

**Conclusion:**

The study highlights a gap between national guidance advocating informed, patient-centred care and women’s lived experiences of miscarriage management in NI. Lack of autonomy and inadequate communication were associated with emotional distress and feelings of disempowerment. Healthcare professionals should ensure women are provided with comprehensive information, sufficient time for decision-making, and genuine choice in treatment whenever medically feasible. Further research is required to examine the long-term psychological impact of different miscarriage management approaches and to inform best practice in compassionate, patient-centred care.

## Background

Miscarriage, also known as spontaneous abortion, is a medical term that indicates the loss of a viable pregnancy. It is the most common gestational complication with a pooled risk of 13–15% of all recognised pregnancies. Further, the prevalence of recurrent miscarriage, two or more losses, is 2–6% [1]. The legal definition of miscarriage varies between countries [2]. In Australia and the USA, miscarriage is defined as a pregnancy loss occurring before the 20th week of the total gestational period of 40 weeks [3], while in the United Kingdom (UK), it is defined as a pregnancy loss occurring before the 24th week of the total gestational period [4].

The range of women’s emotions following miscarriage include grief and sadness [5], isolation and despair [3, 6] as well as reduced overall life satisfaction, resulting in low engagement in social interactions and activities [7]. Many studies describe the development of anxiety or depression following miscarriage [8, 9].

Women who experience symptoms of miscarriage may access different hospital services depending upon the healthcare organisation [10–12]. In the United Kingdom, first trimester miscarriages (up to 13 weeks) are cared for in the Early Pregnancy Assessment Unit (EPAU) [13]. However, some women might not have been known to EPAU services during early weeks of pregnancy and attend the Emergency Department or General Practitioner. Conversely, women who experience late miscarriage (from 13^+ 0^ to 23^+ 6^ weeks) might access services through gynaecology units or maternity wards.

In the United Kingdom, miscarriage can be managed either expectantly, medically, surgically, or with a combination of these treatments, and practitioners are required to follow the NICE guidelines on miscarriage diagnosis and initial management [13]. National Health Service Health and Social Care Trusts in Northern Ireland follow NICE guidelines, which recommends three approaches to the management of miscarriage: expectant, medical, and surgical [13]. However, little is known about how the management of miscarriage influences women’s emotional wellbeing [13, 14]. To date, only a few studies have attempted to investigate the relationship between the management of miscarriage and the development of psychological symptoms [14–17].

Two Cochrane systematic reviews have highlighted the lack of sufficient robust evidence to establish which is the best form of management of miscarriage – medical, surgical or expectant. Thus, when medically stable, women should be given options [18, 19]. Some have suggested that women’s psychological wellbeing may be influenced by their satisfaction with the treatment and not the particular method of management [15]. Others argue that the duration of treatment is linked to emotional outcomes, with shorter treatments – such as medical and surgical – showing lower incidence of emotional distress post-miscarriage [14].

This study explored women’s emotional needs when experiencing miscarriage and attending a hospital facility in NI; including how different approaches to miscarriage management impacted on their emotional wellbeing.

## Methodology

### Aim and objectives

The aim of this study is to examine women’s perceptions of how different approaches to miscarriage management and the support they received in hospital settings impacted on their emotional wellbeing. This study has the following objectives:

- To explore women’s choice of management of miscarriage (expectant, medical and surgical).
- To explore the delivery of information around management of miscarriage.
- To explore the support received by health professionals around women’s management of miscarriage.

### Study design

The study took place between January 2020 and April 2023. It adopted a mixed-methods approach, combining a scoping review of the international literature [20] with analysis of self-reported cross-sectional online survey data from 723 women who had experienced miscarriage, 20 semi-structured interviews with women, and written narrative accounts from 24 health professionals in NI. This manuscript only reports the results from the survey. 

Ethical approval was obtained from the Queen’s University Belfast Faculty of Medicine, Health and Life Sciences Research Ethics Committee on 10th December 2021 (Ref: MHLS 20_99).

### Participants

Women aged over 16 years who had experienced a miscarriage and attended a hospital setting in NI within the previous five years and over the age of 16 years at the time of participation were invited to take part. As gender identity was not queried, all participants were individuals with the biological ability to carry children, and non-birthing partners were not eligible to complete the survey.

### Informed consent and participants’ wellbeing

Participants were provided with an information sheet and consent form before starting the survey and could not continue to complete the survey without providing consent. Due to the sensitive nature of the topic, participants were encouraged to pause or cease completion if they experienced any emotional distress. All participants were provided with a debrief sheet with relevant support contact information so that, in the event of emotional distress, they could reach out.

### Survey

An outline of the survey questionnaire can be viewed in Table 1. The survey was designed in consultation with an advisory group consisting of Health Care Professionals (HCPs) (nurse, gynaecologist, bereavement midwife) and two women with lived experience of miscarriage. It was built and disseminated via the online survey platform Qualtrics (https://www.qualtrics.com) and was ‘live’ for data collection from January 2021 through April 2021.

**Table 1:** Survey structure

1) History of miscarriage
2) Impact of miscarriage
3) Interaction with health professionals while in hospital and provision of information
4) Treatment options decision-making
5) Experiences in different hospital settings
6) Follow-up
7) Overall satisfaction with emotional support received
8) Experience of miscarriage in hospital settings during the Covid-19 pandemic
9) About you - demographics
10) Do you have any suggestions about how your emotional needs might have been better supported in hospital settings, while you were experiencing your miscarriage?
11) How did Covid-19 impact on your experience of miscarriage care in the hospital setting?

Sample characteristics were assessed with single items and included sociodemographic (age, household population, education, and employment status), pregnancy history (number of miscarriages, gestational timeframe of the miscarriage, conception method), and mental health (if participants experienced any mental health issues or had been formally diagnosed with any mental disorders, such as anxiety, depression or PTSD, after miscarriage).

### Sample size calculation

The survey sample size was determined using a standard formula for estimating unknown population parameters from a random sample [21]. At the time of planning, it was assumed that approximately 10–20% of clinically recognized pregnancies resulted in miscarriage annually, corresponding to an estimated 3,000–5,000 cases [22]. Applying this estimated prevalence range to the sample size calculation formula previously used by Pourhoseingholi et al. 2013 [21] indicated that a minimum sample size of between 138 and 240 participants was required [22, 23].

### Data collection

Women were recruited via advertisements on social media (Facebook, Instagram, Twitter) which provided information on the study and a direct link to the survey. The online survey software Qualtrics was used to administer the survey. The survey was designed in consultation with advisory group members and informed by the scoping review [20] and included two screening questions to ensure inclusion and exclusion criteria were met. Women were asked if they attended hospital facilities in NI, in the last five years, when experiencing miscarriage, and if not, they were re-directed to end the survey, and their data was automatically deleted. The survey included needs- and experience-focused questions with nominal (yes/no) and Likert scale response sets.

The survey was divided in nine sections (Table 1) according to a specific topic of interest. Not every question in the survey was applicable to everyone, and each woman was directed to specific questions according to the answer provided. Two open-ended questions were also included in the survey. The first was used to ask women if they had any suggestions on how their emotional needs might have been better supported by health professionals. The second one was related to the effect of Covid-19 on their overall experience of miscarriage care.

### Data analysis

Only data analysis of Sect. 4 (Table 2) is reported in this paper. Other publications have reported on the other sections of the survey [20, 22, 24–26]. The survey was accessed by 1,244 women and fully completed by 723. Only data from fully completed surveys (*N* = 723) were included in the data analysis [27].

**Table 2:** Survey questions

Section 1. Treatment Details		
Question	Response Options / Type	Follow-up / Next Section
What treatment did you have during your miscarriage?	Multiple choice (e.g., Expectant / Medical / Surgical, combination of treatments)	→ Q2
Were you given the opportunity to choose your treatment?	Yes / No	If Yes → Section 1a If No → Section 1b

Section 1a. When You Were Able to Choose Your Treatment		
#	Question	Response Type
1a	Why did you choose this particular treatment? • I wanted my miscarriage to happen naturally • I wanted my miscarriage to happen naturally because of my beliefs • I was afraid of seeing the fetus • I am afraid of surgery • I was afraid of being in pain • I am not sure why I chose this option • My decision was influenced by health professionals • My decision was influenced by family and friends • I previously had a good experience with this treatment • I previously had a bad experience with other treatments • Others (free text)	Tick as many boxes as you like
2a	All treatment options (expectant, medical, surgical) were clearly explained to me by health professionals.	5-point scale: Strongly agree → Strongly disagree
3a	My opinion was considered when deciding on treatment options.	5-point scale
4a	I was given enough information about each treatment option.	5-point scale
5a	I had enough time to think before making my decision.	5-point scale
6a	My feelings were taken into account during discussions about treatment options.	5-point scale
7a	My worries and fears were considered by health professionals.	5-point scale
8a	It was important to me to discuss my treatment options with health professionals.	5-point scale
9a	I felt empowered to discuss my treatment options.	5-point scale

Section 1b. When You Were Not Able to Choose Your Treatment		
#	Question	Response Type
1b	Why were you not able to choose your treatment? • I was very unwell when I arrived in hospital and, therefore, I could not choose my treatment • I become unwell while in hospital and, therefore, I could not choose my treatment • I had an underlying medical condition and, therefore, I could not choose my treatment • Only one treatment was available for my particular situation (i.e. fetal heartbeat detected, premature rupture of membranes before 24 weeks) • Health care professionals chose my treatment, and I do not know why • Other (free text)	Select one of the following statements
2b	My treatment options (expectant, medical, surgical) were clearly explained to me by health professionals.	5-point scale: Strongly agree → Strongly disagree
3b	I had enough time to discuss my treatment with health professionals.	5-point scale
4b	My worries and fears were addressed by health professionals.	5-point scale
5b	I felt upset or angry that I could not choose my treatment.	5-point scale
6b	I understood why I was not able to choose my treatment.	5-point scale

### Quantitative data

Survey results were downloaded from Qualtrics and analysed using SPSS (V26). Responses which contained missing data were excluded from the analysis [27]. Characteristics of the sample such as age; numbers of miscarriages; and type of department where they sought medical care were presented as descriptive statistics. Further variables, such as “Treatment options were clearly explained” and “I had time to discuss my treatment options with health professionals”, were also analysed using descriptive statistics.

### Qualitative data

Question 1a and 1b open-ended questions that allowed space for respondents to explain further. These responses were downloaded from Qualtrics and uploaded into NVivo 11, and content analysis was performed, highlighting codes and themes using different colours. The occurrences of similar words or sentences were grouped in codes and subsequentially organised in themes [28]. Only manifest data analysis was conducted, and themes were not interpreted [29]. Qualitative coding was conducted by MG and discussed among the research team to enhance analytic rigour. All quotes have been anonymised by the use of a code.

### Integration of quantitative and qualitative findings

This manuscript and study were reported in line with the GRAMMS guidance to ensure transparent presentation of mixed-methods design, integration, and interpretation [30]. Integration occurred at the interpretation stage, where qualitative themes were used to explain and extend quantitative trends regarding choice, communication, and emotional outcomes. The thematic framework provided a systematic means of presenting and interpreting the findings. Qualitative data were integrated within the analysis to complement and expand upon the quantitative results, thereby offering a more in-depth understanding of participants’ experiences [31]. An explanatory mixed-methods approach was employed, as this design was deemed most appropriate for elucidating the relationships between the quantitative trends and the qualitative insights, and for providing a comprehensive interpretation of the data [32].

### Findings

The survey was completed fully by 723 respondents. Given the large sample, data included in the analysis are from the *N* = 723 women who completed the full survey using a complete-case approach [33], with partial responses not used.

### Demographic characteristics

Most participants (58%) were between 31 and 39 years old and lived with their partner and children (70%) at the time of the survey. Just under two-thirds (65%) of participants had a higher education qualification and 51% worked in a professional or managerial position. A full description of demographic characteristics can be found in Table 3.

**Table 3:** Demographic characteristics of participants

	*N*=	%
Age		
16–25	48	6.6%
26–30	156	21.6%
31–39	420	58.1%
40–49	99	13.7%
Total	723	100%
Living arrangements		
Live alone	23	3.2%
Partner and other children	507	70.1%
Just partner	191	26.5%
Missing value	1	0.1%
Total	723	100%
Education		
Higher Degree	170	23.5%
BSc Degree	298	41.2%
Higher/A-levels	93	13.1%
Standard grade/GCSE	64	12.9%
No formal qualification	3	0.4%
Total	723	100%
Employment status		
Managerial/professional	370	51.2%
Skilled worker	187	25.9%
Unskilled worker	30	4.1%
Student	23	3.2%
Stay at home parent	68	9.4%
Unemployed	4	0.6%
Other	41	5.7%
Total	723	100%

### History of pregnancy loss and management of miscarriage

Participants’ history of pregnancy loss was explored, and findings indicate that the 43% of the sample had experienced more than 1 miscarriage, with 80% having had a miscarriage during their first trimester. A total of 96% of the sample conceived their pregnancy naturally while 4% utilised assisted reproductive therapies (Table 4).

**Table 4:** History of pregnancy loss and reproductive health

	*N*=	%
Number of miscarriages		
1	414	57.2%
2	153	21.2%
3	83	11.5%
4+	73	10.1%
Total	723	100%
Gestational age at miscarriage		
< 4 weeks	5	0.7%
Between 4 and 6 weeks	92	12.7%
Between 7 and 12 weeks	509	70.4%
Between 13 and 16 weeks	59	8.2%
Between 17 and 20 weeks	38	5.3%
Between 21 and 24 weeks	19	2.6%
Don’t know	1	0.1%
Total	723	100%
Hospital admission		
Yes	262	36.2%
No	461	63.8%
Total	723	100%
Method of conception		
Natural conception	692	95.7%
Assisted reproductive therapies	26	3.6%
Missing	5	0.7%
Total	723	100%

**Fig. 1:**
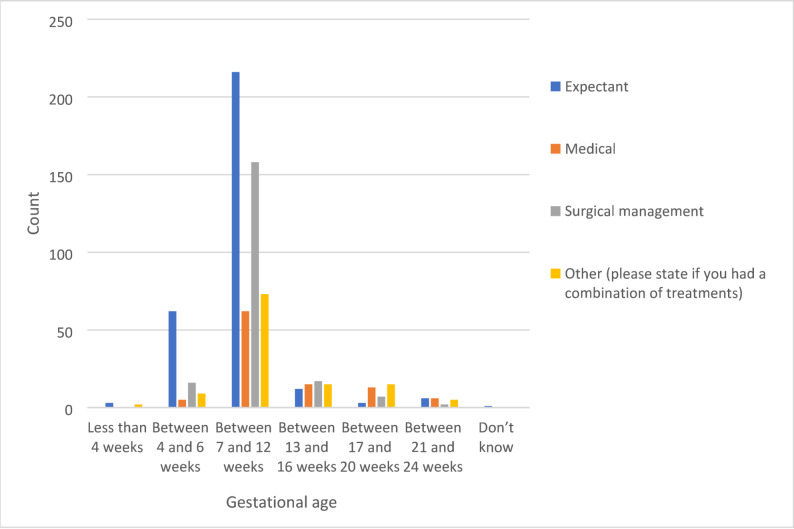
Management of miscarriage and gestational age

Responses indicated that 42% of the total sample had expectant management of miscarriage, 14% medical management, and 28% surgical management, while 16% of women had a combination of treatments (Table 5). Further, Fig. 1 shows the type of management of miscarriage in relation to the gestational age of the fetus.

**Table 5:** Management of miscarriage

		Percentage	Frequency
	Expectant management	42%	304
	Medical management	14%	101
Type of management	Surgical management	28%	202
	Combination of treatments	16%	116
Total		100%	723

### Women’s experience of management of miscarriage

As shown in Tables 6 and 48% of women stated they were able to choose the type of miscarriage management they received (*N* = 344) and the 52% (*N* = 379) said they were not able to do so.

**Table 6:** Women’s opportunity to choose type of miscarriage management

Question	Percentage	Frequency
Women who did not choose treatment	52%	379
Women who choose treatment	48%	344
Total	100%	723

Subsequentially, women were divided in two different groups “Women who were able to choose treatment” and “Women who were not able to choose treatment”. Groups were asked similar questions to gather and insight on their experience. Data were organised in three main themes and 4 main sub-themes. (1) Choice: 1a Health professionals made the decision; 1b Medical reason behind the decision; 1c I was just sent home; 1d I just wanted it to be all over (2) Lack of autonomy in making a decision. (3) Delivery of information: 3a Satisfaction with the delivery of information. (4) Health professional support.

### Theme 1- choice

52% of women (*N* = 379) who did not get a chance to choose how their miscarriage was managed were asked to provide a reason for this (Table 7) while the 48% (*N* = 344) of women who did choose their treatment were asked to provide reason for choosing their treatment (Table 8).

**Table 7:** Reasons why women were not able to choose their treatments

Why were you not able to choose your treatment?	Frequency	Percentage
I was very unwell when I arrived in hospital and, therefore, I could not choose my treatment	27	7.1%
I become unwell while in hospital and, therefore, I could not choose my treatment	9	2.4%
I had an underlying medical condition and, therefore, I could not choose my treatment	3	0.8%
Only one treatment was available for my particular situation (i.e. fetal heartbeat detected, premature rupture of membranes before 24 weeks)	68	17.9
Health care professionals chose my treatment, and I do not know why	138	36.4%
Others (open text):	117	31%
Treatment not available due to Covid	17	
“Just sent home”	17	
Women’s choice not considered	4	
No offer was ever made	9	
Staff-hospital facility not available	3	
Miscarriage already took place before visiting hospital	48	
Medical condition/ reason behind it	19	
Total	379	100%

**Table 8:** Reasons why women choose a particular treatment

Why did you have this particular treatment?	Frequency	Percentage
I wanted my miscarriage to happen naturally	102	14.2%
I wanted my miscarriage to happen naturally because of my beliefs	13	1.8%
I was afraid of seeing the fetus	39	5.4%
I am afraid of surgery	28	3.9%
I was afraid of being in pain	27	3.8%
I am not sure why I chose this option	17	2.4%
My decision was influenced by health professionals	92	12.8%
My decision was influenced by family and friends	26	3.6%
I previously had a good experience with this treatment	15	2.1
I previously had a bad experience with other treatments	14	2.0%
Others (Open text):	74	16.5%
Medical reasons	25	
Covid-19	2	
“*I just wanted it all over*”	22	
Did not want to miscarry at home/ be away with children	9	
Afraid of surgery/did not want surgery	3	
Burial rituals; genetic test	2	
*“I hoped they were wrong”*	1	
Miscarriage happened naturally	1	
No need for intervention	1	
Most effective intervention for missed miscarriage	1	
Advice from others	2	
Avoid pain	1	

#### Sub-theme 1a. health professionals made the decision

Some women who were not able to choose their treatment said that HCPs chose on their behalf, and they did not know the reason behind it. The following quote is representative of women’s testimonies.

“*I wasn’t given any choice of how to manage the miscarriage. I feel like I was failed by the NHS. Both my miscarriages took three to four weeks from the first bleeding to being confirmed as complete. I was going to work afraid I would miscarry at any moment. It was awful*” (SP1).

#### Sub-theme 1b. medical reasons behind the decision

Many women explained that, for a medical reason, they could not choose their treatment.

*“Baby estimated death was 8–10 weeks old due to size*,* due to missed miscarriage body had not expelled baby so medical management was first option and if it didn’t work then surgical” (SP2).*

“*I had been having extreme bleeding for ten days before miscarrying. Baby was fine until then. So*,* they did surgical procedure as they wanted to keep blood loss under control*” (SP3).

#### Sub-theme 1c. I was just sent home

Some women were discharged home by health care professionals after being diagnosed with a miscarriage. These are some of the women’s testimonies which are representative of their experience.

*“The only treatment I received was bloods taken*,* confirmed that my hormone level was lower than it should be and sent home to come back the following day for a scan. In that time I naturally miscarried” (SP13)*.

*“I was sent home to await natural miscarriage” (SP4)*.

#### Sub-theme 1d I just wanted it to be all over

Some women who were able to choose their treatment explained that they wanted a quick resolution of their miscarriage to avoid further delays or emotional distress

“[…] I chose surgical management to get the process over and quickly; I felt that the surgery would be less painful and emotional for me” (SP5).

Others explained that the reason behind a quick resolution of their miscarriage was that they did not want to miscarry at home around their children, and they wished to resolve the miscarriage quickly so they could get back to their family or work.

I did not want my young kids to witness miscarrying at home naturally, I didn’t want the baby to be flushed down the toilet *(SP14)*.

### Theme 2 lack of autonomy in making a decision

Women who were not able to choose how their miscarriage was managed were asked how they felt about not having been given a choice. While 25% of them said they were upset and angry, 42% remained neutral. Conversely, the 84% of women who were able to choose their treatment explained that having a choice was important for them. Table 9 outlines women’s responses and the following quotes are representative of their experiences.

“*I was clear about wanting a D&C but was told I’d have to go away and think about it*,* come back for a covid test after the weekend and then come back and wait and see if they could fit me in but there were no guarantees. I explained I was petrified of losing naturally and I wasn’t listened to*,* those days at home afterwards were excruciating*” (SP6).

*“Everyone’s feelings should be respected even if they clearly don’t match well with that of the midwife on duty” (SP7)*.

**Table 9:** The importance of choosing

Women who did not choose their treatment			Women who did choose their treatment		
I was upset or angry I could not choose my treatment			It was important to me to discuss my treatment options with HCPs		
Strongly agree	48	12.7%	Strongly agree	203	59.0%
Somewhat agree	48	12.7%	Somewhat agree	85	24.7%
Neither agree or disagree	159	42.0%	Neither agree or disagree	47	13.7%
Somewhat disagree	52	13.7%	Somewhat disagree	7	2.0%
Strongly disagree	72	19%	Strongly disagree	2	0.6%
Total	379	100%	Total	334	100%

### theme 3- delivery of information

#### Satisfaction with the delivery of information

The 40% of women who did not choose their management of miscarriage were satisfied with the amount of information received about treatment options. Conversely, 40% did not receive clear information about treatment options and 20% remained neutral. Full responses can be found in Tables 10, and the following quotes illustrate experiences:

*“When I asked the midwife for advice about the D&C she said that she doesn’t know anything about the procedure and couldn’t advise me. I asked her advice in an attempt to engage with her more*,* I was surrounded by male doctors poking and prodding at me throughout the night and I just wanted some guidance and compassion from the midwife. Unfortunately*,* I did not experience that.*” (SP8).

Some women explained they were sent home without being given information about the different options available (expectant, medical, surgical) or what to expect.

*“I was sent away with no idea what to expect. The dr [doctor] didn’t even look at the amount of blood I was losing” (SP9)*.

*“I was told at the 12 weeks scan the baby had not developed. I was not made aware that [there] were management options. I had another scan at 14 weeks*,* and nothing had changed. I was sent home with no useful information. My friend then called on my behalf and insisted on treatment. I was then offered surgical management” (SP10)*.

In contrast, among the women who chose their treatment, the 83% of respondents received clear information about management of miscarriage while 10% said that they did not receive enough information and another 7% remained neutral.

**Table 10:** Delivery of information about the treatment of miscarriage

Women who did not choose their treatment			Women who did choose their treatment		
During my hospital visit, my treatment options were clearly explained to me by HCPs			During my hospital visit, my treatment options were clearly explained to me by HCPs		
Strongly agree	74	19.5%	Strongly agree	183	53.2%
Somewhat agree	81	21.4%	Somewhat agree	102	29.7%
Neither agree or disagree	73	19.3%	Neither agree or disagree	22	6.4%
Somewhat disagree	70	18.5%	Somewhat disagree	26	6.4%
Strongly disagree	81	21.4%	Strongly disagree	11	7.6%
Total	379	100%	Total	344	100%

### Theme 4- health professional support

The 44% of women in the women who did not chose treatment said that they did not have enough time to discuss their treatment option with HCPs, 33% of women stated that they did have enough time and 23% remained neutral. Conversely, 76% of those in the “Women who chose treatment” group were given enough time to think about their treatment while 14% thought they did not have enough time and 10% remained neutral. Full responses are illustrated in Table 11.

*“I feel like more time needs to be given before options are explained in regard to miscarrying. You are told this heartbreaking news then they talk to you about options etc. when your mind just isn’t able to function properly” (SP11)*.

*“Felt I could have been given a little more time to understand my options*,* I was asked immediately and had to give an answer or whether I wanted surgical or medical. That’s a big decision” (SP12)*.

**Table 11:** Satisfaction with the time spent with HCPs to discuss treatment options

Women who did not choose their treatment			Women who did choose their treatment		
I had enough time to discuss my treatment with HCPs			I was given enough time to think about my treatment options before making my decision		
Strongly agree	53	14.0%	Strongly agree	169	49.1%
Somewhat agree	71	18.7%	Somewhat agree	95	27.6%
Neither agree or disagree	85	22.4%	Neither agree or disagree	33	9.6%
Somewhat disagree	82	21.6%	Somewhat disagree	25	7.3%
Strongly disagree	88	23.2%	Strongly disagree	22	6.4%
Total	379	100%	Total	344	100%

## Discussion

This study aimed to explore women’s perceptions of how different approaches to miscarriage management and the support they received in hospital settings impacted their emotional wellbeing. The aim and objectives of the study were met. This study provided insight into women’s experiences of different miscarriage treatments and highlighted that some women may not have been able to choose their treatment or be involved in the decision-making process, with healthcare professionals making the decision without providing a reason. Additionally, this study indicates that women are not always aware of the three different approaches to miscarriage management (expectant, medical, and surgical) suggesting that the provision of information about miscarriage management may not always be adequate. Finally, women indicated that they were not always satisfied with the time spent with healthcare professionals discussing their different treatment options.

### Choice of management of miscarriage

To the best of our knowledge, no other studies have examined the perceived impact of miscarriage management on women’s emotional wellbeing in Northern Ireland. Some women in this study described being sent home without discussion of alternatives, leaving them disempowered. Broader maternity research in Northern Ireland also shows that women are not consistently offered choice in services or birth settings, with positive experiences linked to shared decision-making with health professionals [34, 35]. National Health Service Health and Social Care Trusts in Northern Ireland follow NICE guidelines, which recommends three approaches to the management of first trimester miscarriage: expectant, medical, and surgical [13]. All options should be available to women, with care tailored to gestational age for example, those up to 13 weeks of the total are generally managed in early pregnancy units. However, it is important to note that an option may not be available to women due to either the gestational age of the fetus or a medical emergency. In these scenarios, it is important to communicate the care plan to women and explaining the reasons why a treatment option may or may not be available to them.

External factors also shape care. During the Covid-19 pandemic, elective surgical procedures, including miscarriage management, were restricted [25, 36]. Another possible explanation for these findings may be that women were not medically stable when attending hospital settings or at risk of bleeding [13] and health professionals chose a treatment approach for them but did not fully explain it. Conversely, it has also been reported that, depending on different factors such as clinical context, health professionals’ preferences and resources, women are not always offered the full range of treatments [37, 38].

### Delivery of information

Delivery of information is part of any health professional’s main duties. In fact, not only do HCPs have a duty of care to sensitively communicate information about their care to patients, but they also have to provide sufficient information to patients to enable them to make informed decisions about their care [39]. Moreover, the delivery of appropriate information to patients contributes to maintaining their autonomy and dignity [40]. It is a patient’s right to receive information about their care and be educated about it [41]. In this light, health professionals should provide accurate and extensive information to patients to ensure their needs are met.

Despite the reasoning behind health professionals either deciding on behalf of women or not providing information on management of miscarriage, patients should be explicitly informed of the reasons for the choices made [42]. It is important to women that they are offered a choice, where possible, rather than just being pointed to expectant management [43]. A relatively recent study conducted in Australia showed how after the introduction of medical management fewer women chose to miscarry expectantly [44].

### Support received in hospital

Some suggest that women’s psychological wellbeing may be influenced by their satisfaction with the treatment, rather than on a particular method of management [15]. The emotional response of early miscarriage does not differ between women who have expectant and those who have medical management of miscarriage [45]. Others argue that the duration of treatment is linked to emotional outcomes, with shorter treatments, such as medical and surgical, showing lower incidence of emotional distress post-miscarriage [14]. Women who choose surgical management might be driven by a rapid resolution of symptoms and the need to return to a “sense of normality” [17]. However, expectant management, for example, might provide more time for a woman to accept the diagnosis of miscarriage, and might be more suitable for those who have difficulties in accepting a failed pregnancy [38].

Regardless of the type of management chosen, there is a need for women to have more time and information to reflect on their choices [46–48]. These findings were in line with previous research, which showed how women who did not have the chance to choose their treatment felt they did not have enough time to discuss treatment options with HCPs. Many reported that HCPs chose their treatment without providing any information about the reason why that decision had been made.

### Implications for practice and research

One of the main principles of patient- centred care is that patients are directly involved in making decisions about their care [39]. For instance, women who prefer surgical management should have this option available [49]. However, around 52% of women in this research study claimed they could not choose treatment. Some of the resources available to both HCPs and patients in Northern Ireland that are used to inform women about management of miscarriage adopt a clear language about treatment “*choice*” [50]. Nevertheless, as revealed in this study, some could argue that women are not equipped to make an informed decision if they have not been provided with adequate information about the different options available to manage a miscarriage. The emotional impact of the lack of choice on parents is unknown. However, ‘choice’ within healthcare is a fundamental element of high-quality care, as is widely supported by the literature and best practice guidelines [42, 51, 52]. In the absence of medical reasons which clearly advise a woman against a certain treatment, they should be presented with detailed information of all three options [18]. Sexual and reproductive health is considered a human right, and States are obliged to respect, protect, and fulfil rights related to women’s sexual and reproductive health. These services include accessing high quality services [53]. The Beijing Platform for Actions clearly stated that it is a human right for women to have control over and decide on their sexual and reproductive health [53].

### Strengths and limitations

To the best of our knowledge this is the first study conducted in Northern Ireland that explored women’s experiences of the management and is one of few internationally. It provides an insight into women’s experiences of miscarriage management, combining quantitative survey data with rich qualitative accounts, which allows for a nuanced understanding of both treatment choices and the emotional impact of care. The relatively large sample size (*N* = 723) enhances the reliability of descriptive statistics, while subgroup analyses, such as women who were able versus unable to choose their treatment, highlight disparities in care and information provision. Including open-ended responses and direct quotations adds depth, giving voice to participants and illustrating real-world implications of policy and clinical practice.

The data were collected retrospectively, which may introduce recall bias and affect the accuracy of women’s reported experiences. The self-selected sample could lead to response bias, with those having stronger opinions or negative experiences more likely to participate. Additionally, the study design is observational, limiting the ability to infer causal relationships between type of management, choice, and psychological outcomes. Finally, because recruitment occurred online and the sample had a higher education and employment profile than the general NI population, findings may not fully represent all women who experience miscarriage.

## Data Availability

Data are available upon reasonable request. If there is a reasonable request, deidentified participant data used in the research are available via emailing the corresponding author after publication.

## References

[1] Quenby S, Gallos ID, Dhillon-Smith RK, Podesek M, Stephenson MD, Fisher J, et al. Miscarriage matters: the epidemiological, physical, psychological, and economic costs of early pregnancy loss. Lancet. 2021;397:1658–67. 10.1016/S0140-6736(21)00682-6. 33915094 10.1016/S0140-6736(21)00682-6

[2] Kerstin A, Wanger B. Complicated grief after perinatal loss. Dialogues Clin Neurosci. 2012;14:187–94. 22754291 10.31887/DCNS.2012.14.2/akersting PMC3384447

[3] Bellhouse C, Temple-Smith M, Watson S, Bilardi J. The loss was traumatic… some healthcare providers added to that: Women’s experiences of miscarriage. Women Birth. 2019;32:137–46. 10.1016/j.wombi.2018.06.006. 30153984 10.1016/j.wombi.2018.06.006

[4] RcOG. The Investigation and Treatment of Couples with Recurrent First-trimester and Second-trimester Miscarriage. RCOG Green-top Guideline 17. 2011;1–18. 10.4103/2230-8210.107834.

[5] Keefe-Cooperman K, A Comparison Of Grief As Related. To Miscarriage And Termination For fetal Abnormality. 2004.

[6] Volgsten H, Jansson C, Svanberg AS, Darj E, Stavreus-Evers A. Longitudinal study of emotional experiences, grief and depressive symptoms in women and men after miscarriage. Midwifery. 2018;64:23–8. 10.1016/j.midw.2018.05.003. 29864578 10.1016/j.midw.2018.05.003

[7] Huss B. Well-Being Before and After Pregnancy Termination: The Consequences of Abortion and Miscarriage on Satisfaction With Various Domains of Life. J Happiness Stud. 2021;22:2803–28. 10.1007/s10902-020-00350-5.

[8] Farren J, Mitchell-Jones N, Verbakel JY, Timmerman D, Jalmbrant M, Bourne T. The psychological impact of early pregnancy loss. Hum Reprod Update. 2018;24:731–49. 10.1093/humupd/dmy025. 30204882 10.1093/humupd/dmy025

[9] Farren J, Jalmbrant M, Falconieri N, Mitchell-Jones N, Bobdiwala S, Al-Memar M, et al. Posttraumatic stress, anxiety and depression following miscarriage and ectopic pregnancy: a multicenter, prospective, cohort study. Am J Obstet Gynecol. 2020;222:367.e1-367.e22. 10.1016/j.ajog.2019.10.102. 10.1016/j.ajog.2019.10.102 31953115

[10] Larivière-Bastien D, deMontigny F, Verdon C. Women’s Experiences of Miscarriage in the Emergency Department. J Emerg Nurs. 2019;45:670–6. 10.1016/j.jen.2019.06.008. 31495508 10.1016/j.jen.2019.06.008

[11] Merrigan JL, Educating Emergency Department Nurses About Miscarriage. MCN, Am J, Matern Child. Nurs. 2018;43:26–31. 10.1097/NMC.0000000000000391. 10.1097/NMC.0000000000000391 29215421

[12] Norton W, Furber L. An exploration of how women in the UK perceive the provision of care received in an early pregnancy assessment unit: An interpretive phenomenological analysis. BMJ Open. 2018;8. 10.1136/bmjopen-2018-023579. 10.1136/bmjopen-2018-023579 PMC6104788 30121616

[13] National Institute for Health and Care Excellence (NICE). Ectopic pregnancy and miscarriage: diagnosis and initial management | NICE guideline [NG126]. 2019. 35077089

[14] Volgsten H, Jansson C, Darj E, Stavreus-Evers A. Women’s experiences of miscarriage related to diagnosis, duration, and type of treatment. Acta Obstet Gynecol Scand. 2018;97:1491–8. 10.1111/aogs.13432. 30063247 10.1111/aogs.13432

[15] Kong GWS, Lok IH, Yiu AKW, Hui ASY, Lai BPY, Chung TKH. Clinical and psychological impact after surgical, medical or expectant management of first-trimester miscarriage - A randomised controlled trial. Aust N Z J Obstet Gynaecol. 2013;53:170–7. 10.1111/ajo.12064. 23488984 10.1111/ajo.12064

[16] Olesen ML, Graungaard AH, Husted GR. Deciding treatment for miscarriage–experiences of women and healthcare professionals. Scand J Caring Sci. 2015;29:386–94. 10.1111/scs.12175. 25236762 10.1111/scs.12175

[17] Ogden J, Maker C. Expectant or Surgical Management of Miscarriage: A Qualitative Study. Obstet Gynecol Surv. 2004;59:585–7. 10.1097/01.OGX.0000134503.29426.DA. 10.1111/j.1471-0528.2004.00121.x 15104611

[18] Kim C, Barnard S, Neilson JP, Hickey M, Vazquez JC, Dou L. Medical treatments for incomplete miscarriage. Cochrane Database of Systematic Reviews. 2017;2017. 10.1002/14651858.CD007223.PUB4/MEDIA/CDSR/CD007223/IMAGE_N/NCD007223-CMP-008-07.PNG. 10.1002/14651858.CD007223.pub4 PMC6464743 28138973

[19] Nanda K, Lopez LM, Grimes DA, Peloggia A, Nanda G. Expectant care versus surgical treatment for miscarriage. Cochrane Database Syst Reviews. 2012. 10.1002/14651858.cd003518.pub3. 10.1002/14651858.CD003518.pub2 16625583

[20] Galeotti M, Mitchell G, Tomlinson M, Aventin Á. Factors affecting the emotional wellbeing of women and men who experience miscarriage in hospital settings: a scoping review. BMC Pregnancy and Childbirth 2022 22:1. 2022;22:1–24. 10.1186/S12884-022-04585-3. 10.1186/s12884-022-04585-3 PMC8974061 35361132

[21] Pourhoseingholi MA, Vahedi M, Rahimzadeh M. Sample size calculation in medical studies. Gastroenterol Hepatol Bed Bench. 2013;6:14–7. 10.22037/ghfbb.v6i1.332. 24834239 PMC4017493

[22] Galeotti M. The emotional needs of women who experience miscarriage in hospital settings: a mixed-methods needs assessment in Northern Ireland. Queen’s University Belfast; 2023. https://pure.qub.ac.uk/en/studentTheses/the-emotional-needs-of-women-who-experience-miscarriage-in-hospit/.

[23] Galeotti M, Robinson M, Mitchell G, Tomlinson M, White J, Aventin Á. An online survey of women’s perceived care needs following miscarriage in hospital settings in Northern Ireland. 2023. 10.21203/RS.3.RS-2718563/V1.

[24] Aventin A, Robinson M, White J, Galeotti M. Recurrent pregnancy loss, psychological distress and wellbeing support for women: a mixed-methods analysis. BMC Womens Health. 2025;25. 10.1186/s12905-025-04079-2. 10.1186/s12905-025-04079-2 PMC12581269 41184880

[25] Heaney S, Galeotti M, Aventin Á. Pregnancy loss following miscarriage and termination of pregnancy for medical reasons during the COVID-19 pandemic: a thematic analysis of women’s experiences of healthcare on the island of Ireland. BMC Pregnancy Childbirth. 2023;23:1–10. 10.1186/S12884-023-05839-4/TABLES/2. 37480006 10.1186/s12884-023-05839-4 PMC10360341

[26] Robinson M, Galeotti M, Mitchell G, Tomlinson M, Aventin Á. Network analysis and comparison of psychological distress among women with miscarriage experience. Psychol Health Med. 2025. 10.1080/13548506.2025.2587261. 41275503 10.1080/13548506.2025.2587261

[27] San Lazaro Campillo I, Meaney S, Sheehan J, Rice R, O’Donoghue K. Reproductive Health Knowledge About Miscarriage: A Cross-Sectional Study of University Students. Matern Child Health J. 2021;25:282–92. 10.1007/s10995-020-03017-y. 33190195 10.1007/s10995-020-03017-y

[28] Hsieh H-F, Shannon SE. Three Approaches to Qualitative Content Analysis. 2005. 10.1177/1049732305276687. 10.1177/1049732305276687 16204405

[29] Bengtsson M. How to plan and perform a qualitative study using content analysis. NursingPlus Open. 2016;2:8–14. 10.1016/j.npls.2016.01.001.

[30] O’Cathain A, Murphy E, Nicholl J. The quality of mixed methods studies in health services research. J Health Serv Res Policy. 2008;13:92–8. 18416914 10.1258/jhsrp.2007.007074

[31] Ziegler N, Kang L. Mixed methods desings. In: Moeller AJ, Creswell JW, Saville N, editors. Second Language Assessment and Mixed Methods Research. Camridge: Cambridge University Press; 2016.

[32] Creswell JW, Klassen AC, Plano Clark VL, Clegg Smith K. Best Practices for Mixed Methods Research in the Health Sciences. 2011.

[33] Ross RK, Breskin A, Westreich D. When Is a Complete-Case Approach to Missing Data Valid? The Importance of Effect-Measure Modification. Practice of Epidemiology. 2020;189:1583–9 10.1093/aje/kwaa124. 10.1093/aje/kwaa124 PMC7705610 32601706

[34] Alderdice F, Hamilton K, Mcneill J, Lynn F, Curran R, Redshaw M, Birth NI. A Survey of Women’s Experience of Maternity Care in Northern Ireland. Belfast; 2016. https://research.hscni.net/publication/birth-ni-survey-womens-experience-maternity-care-northern-ireland-2016.

[35] Watkins V, Nagle C, Kent B, Street M, Hutchinson AM. Labouring Together: Women’s experiences of Getting the care that I want and need in maternity care. Midwifery. 2022;113. 10.1016/j.midw.2022.103420. 10.1016/j.midw.2022.103420 35849913

[36] Silverio SA, De Backer K, Easter A, von Dadelszen P, Magee LA, Sandall J. Women’s experiences of maternity service reconfiguration during the COVID-19 pandemic: A qualitative investigation. Midwifery. 2021;102. 10.1016/j.midw.2021.103116. 10.1016/j.midw.2021.103116 PMC9756856 34399382

[37] Dalton VK, Harris LH, Clark SJ, Cohn L, Guire K, Fendrick AM. Treatment Patterns for Early Pregnancy Failure in Michigan. https://home.liebertpub.com/jwh. 2009;18:787–93. 10.1089/JWH.2008.1091 10.1089/jwh.2008.1091 PMC2851131 19445643

[38] Shorter JM, Atrio JM, Schreiber CA. Management of early pregnancy loss, with a focus on patient centered care. Semin Perinatol. 2019;43:84–94. 10.1053/j.semperi.2018.12.005. 30739750 10.1053/j.semperi.2018.12.005

[39] NMC. (Nursing and Midwifery Council). The Code. 2018.

[40] Gerteis M, Edgman-Levitan S, Daley J, Delbanco TL. Through the patient’s eyes: understanding and promoting patient-centered care. Jossey-Bass; 2002.

[41] Sripad P, Merritt MW, Kerrigan D, Abuya T, Ndwiga C, Warren CE. Determining a Trusting Environment for Maternity Care: A Framework Based on Perspectives of Women, Communities, Service Providers, and Managers in Peri-Urban Kenya. Front Glob Womens Health. 2022;0:41. 10.3389/FGWH.2022.818062. 10.3389/fgwh.2022.818062 PMC9069110 35528311

[42] Victoor A, Delnoij DM, Friele RD, Rademakers JJ. Determinants of patient choice of healthcare providers: A scoping review. BMC Health Serv Res. 2012;12:1–16. 10.1186/1472-6963-12-272/TABLES/2. 22913549 10.1186/1472-6963-12-272 PMC3502383

[43] Petrou S, McIntosh E. Women’s Preferences for Attributes of First-Trimester Miscarriage Management: A Stated Preference Discrete-Choice Experiment. Value Health. 2009;12:551–9. 10.1111/J.1524-4733.2008.00459.X. 18798807 10.1111/j.1524-4733.2008.00459.x

[44] Black KI, de Vries BS, Moses F, Pelosi M, Cong A, Ludlow J. The impact of introducing medical management on conservative and surgical management for early pregnancy miscarriage. Aust N Z J Obstet Gynaecol. 2017;57:93–8. 10.1111/AJO.12573. 28251638 10.1111/ajo.12573

[45] Fernlund A, Jokubkiene L, Sladkevicius P, Valentin L, Sjöström K. Psychological impact of early miscarriage and client satisfaction with treatment: comparison between expectant management and misoprostol treatment in a randomized controlled trial. Ultrasound Obstet Gynecol. 2021;58:757–65. 10.1002/UOG.23641. 33798287 10.1002/uog.23641

[46] Kong GWS, Chung TKH, Lok IH. The impact of supportive counselling on women’s psychological wellbeing after miscarriage–a randomised controlled trial. BJOG. 2014;121:1253–62. 10.1111/1471-0528.12908. 24912398 10.1111/1471-0528.12908

[47] Linnet Olesen M, Graungaard AH, Husted GR. Deciding treatment for miscarriage - experiences of women and healthcare professionals. Scand J Caring Sci. 2015;29:386–94. 10.1111/scs.12175. 25236762 10.1111/scs.12175

[48] Lariviere-Bastien D, DeMontigny F, VC. Women’s experiences of miscarriage in the emergency department. J Emerg Nurs. 2019;45:670–6. 31495508 10.1016/j.jen.2019.06.008

[49] Westhoff CL, A Better Medical Regimen for the Management of Miscarriage. N Engl J Med. 2018;378. 10.1056/NEJMe1803491. 10.1056/NEJMe1803491 29874544

[50] Belfast Health &. Social Care Trust. Your choices for treatment of miscarriage. 2020.

[51] Gabe J, Harley K, Calnan M. Healthcare choice: Discourses, perceptions, experiences and practices: http://dx.doi.org/101177/0011392115590061. 2015;63:623–35. 10.1177/0011392115590061

[52] Aalto AM, Elovainio M, Tynkkynen LK, Reissell E, Vehko T, Chydenius M, et al. What patients think about choice in healthcare? A study on primary care services in Finland. Scand J Public Health. 2018;46:463–70. 10.1177/1403494817731488. 28925813 10.1177/1403494817731488

[53] United Nations. OHCHR | Sexual and reproductive health and rights. 2022. https://www.ohchr.org/en/women/sexual-and-reproductive-health-and-rights. Accessed 9 Nov 2022.

